# A deterministic method for quantifying spindle-shaped cells in noisy bright-field microscopy

**DOI:** 10.1038/s41598-026-51592-x

**Published:** 2026-05-21

**Authors:** Martin Radvanský, Markéta Vašinková, Miloš Kudělka, Eva Kriegová, Petr Gajdoš

**Affiliations:** 1 Department of Computer Science, FEECS, VSB - Technical University of Ostrava, 17. listopadu 2172/15, 70800 Ostrava, Czech Republic; 2 Department of Immunology, Faculty of Medicine and Dentistry, Palacky University & University Hospital, Hněvotínská 976/3, 775 15 Olomouc, Czech Republic

**Keywords:** Cell counting, Fibroblast-like cells, Spindle-shaped cells, Bright-field microscopy, Contour detection, Gaussian-based model, Biological techniques, Cell biology, Computational biology and bioinformatics, Mathematics and computing

## Abstract

Accurate quantification of spindle-shaped cells in bright-field microscopy remains challenging due to low contrast, noise, and highly variable cell morphology. Conventional approaches often rely on fluorescent staining or deep learning models, which may introduce phototoxic effects, require extensive training data, or offer limited interpretability. Here, we present a deterministic image analysis method for quantifying spindle-shaped cells directly from noisy bright-field microscopy images without the need for fluorescent labeling or supervised training. The proposed workflow combines contrast enhancement, adaptive thresholding, contour filtering, and shape-guided refinement to detect elongated cell bodies under challenging imaging conditions. The method is designed to be robust to irregular cell morphology and heterogeneous background commonly encountered in bright-field time-lapse microscopy. Quantitative evaluation demonstrates reliable cell counting and consistent performance across varying noise levels and imaging conditions. The approach achieves competitive accuracy while maintaining interpretability and low computational complexity, enabling straightforward integration into existing biomedical imaging workflows. By preserving cell morphology and avoiding fluorescent staining and complex model training, the method provides a practical solution for large-scale analysis of spindle-shaped cell populations in bright-field microscopy experiments.

## Introduction

**Fig. 1:**
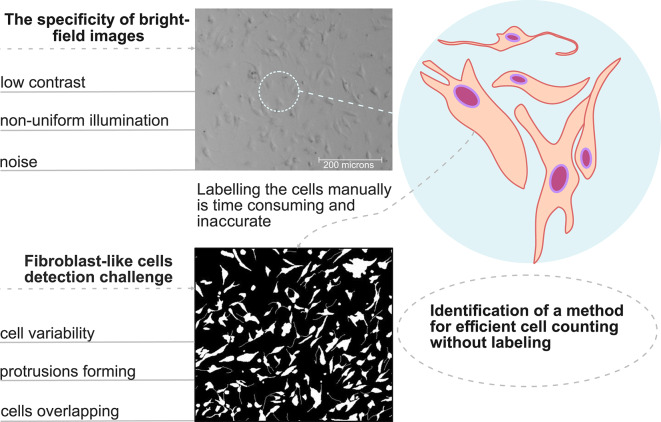
Specificity of fibroblasts and fibroblast-like cells in bright-field microscopy images from time-lapse devices.

Automated cell detection, quantification, or counting is a critical tool in many areas of biological and medical research, as it facilitates the analysis of cellular behavior in dynamic and complex environments^1^. While automated cell counting from fluorescent and high-contrast microscopy images has become widely accessible, thanks to advances in artificial intelligence, image processing, and a range of user-friendly tools, bright-field microscopy still poses significant challenges, particularly due to low contrast and high levels of noise^2,3^. These challenges are exacerbated when images contain cells with variable and irregular shapes, which complicates their separation from the background (Fig. 1).

In low-contrast imaging modalities such as bright-field, phase contrast, and differential interference contrast, identifying cell boundaries typically requires fluorescent staining or the use of fluorescent proteins^4^. However, fluorescent dyes can be toxic to living cells^5^ and substantially increase experimental costs. For example, a 5 mL vial of 20 mmol/L Hoechst 33342 solution costs approximately $130–150 (https://www.thermofisher.com/order/catalog/product/62249), making large-scale applications financially prohibitive, especially in resource-limited laboratories. These additional expenses become particularly burdensome in high-throughput studies, highlighting the need for more affordable and non-invasive alternatives.

Furthermore, manual labeling of cells in microscopy images is often unreliable due to human error and image complexity. High inter-observer and intra-observer variability are common: different annotators may label the same cells differently, and even the same annotator may be inconsistent over time^6^. Factors such as fatigue, subjectivity, and the difficulty of interpreting overlapping or low-contrast cells can significantly affect the accuracy and reproducibility of manual annotations^7,8^. In fact, some studies report that automated algorithms outperform human annotators by detecting cells that were otherwise overlooked due to human error^9^.

**Fig. 2:**
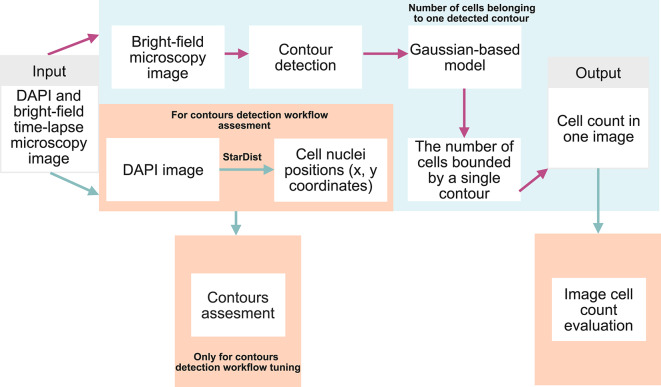
The development of our method is based on time-lapse microscopy images of fibroblast-like synoviocytes. We utilize grayscale bright-field images, where the primary objective is to determine the number of cells, and corresponding fluorescent (DAPI) images of the same cells, in which the nuclei are stained. The fluorescent images serve as a ground truth reference to validate the accuracy of the image processing step that aims to extract cell contours (orange). This contour extraction step is a critical component of the overall workflow and relies on conventional image processing techniques to approximate individual cell boundaries. The process performed by the user of our workflow does not include these annotations; therefore, the resulting pipeline involves only selected steps (marked in blue).

Fibroblasts, in particular, present a considerable detection challenge due to their unstable, irregular morphology and frequent overlap with surrounding structures. Nevertheless, tracking their behavior is essential for studying responses to environmental stimuli or pharmacological treatments^10^. In this study, we propose an automated method for cell counting in grayscale bright-field microscopy images that eliminates the need for fluorescent labeling. Our approach combines traditional image processing techniques with statistical methods to accurately estimate both the number and positions of cells. This work not only improves the accessibility of cell quantification in label-free imaging conditions but also lays the groundwork for future developments in cell tracking based on these complex image data. The proposed workflow is illustrated in Fig. 2. The scheme clearly illustrates that fluorescent-labeled images are used solely for the development of the workflow for assessment and evaluation; they are not required by the end users.

### Automated counting and detection of fibroblasts and fibroblast-like cells

Fibroblasts are mesenchymal cells residing in connective tissues, where they produce extracellular matrix (ECM) and collagen and regulate surrounding cell types^11–13^. Their motility and contractility are key to tissue remodeling and wound healing^14^. Fibroblasts display diverse morphologies depending on their activation state^15^, microenvironment^16^, and tissue context^17^. Fibroblast-like cells share similar morphology but originate from different lineages^18^, such as tumor cells in synovial sarcoma lines with spindle-shaped features^19^. Due to shared morphology and behavior, our method applies to both fibroblasts and fibroblast-like cells.

In standard 2D cultures on glass or plastic, fibroblasts and fibroblast-like cells adhere well and are suitable for live-cell imaging and migration studies^20^, while their rapid proliferation makes them ideal for basic cell biology research^21^.

Detecting or segmenting these cells in microscopy images is challenging due to their variable shapes, influenced by activation, environment, or tissue type^15–17^. Upon activation, fibroblasts often shift from a spindle to a stellate shape^22^, contributing to tissue heterogeneity and diagnostic complexity^23–25^. In complex tissues like tumors or fibrotic lesions, distinguishing them from neighboring cells is difficult^26,27^. Variability in morphology and the presence of imaging artifacts further hinder detection. Although machine learning methods help, they require large annotated datasets, which are often lacking in biomedical contexts.

### Cell segmentation vs. cell detection

Cell detection and segmentation are fundamental steps in microscopy image analysis and form the basis for tasks such as phenotype classification, counting, and tracking in time-lapse microscopy. In computer vision, detection involves localizing and classifying objects using bounding boxes, while semantic segmentation assigns a label to each pixel^28,29^. Instance segmentation further distinguishes between individual objects of the same class, integrating both localization and semantic labeling^30,31^.

In microscopy, the objects of interest are typically cells or cell nuclei. Regardless of the specific task, be it counting, classification, or tracking, the accurate localization of each cell is essential. Unlike general-purpose object detection, cell segmentation often requires resolving touching or overlapping objects. This makes instance-level methods especially relevant, including both traditional image processing techniques and modern deep learning approaches based on convolutional neural networks^32^. However, in bright-field microscopy, particularly under low-contrast and noisy conditions, deep learning methods may not always be feasible due to the need for large annotated datasets and high computational costs. Additionally, the morphological variability of cells (e.g. fibroblast and fibroblast-like cells) and the presence of multiple cell types or phenotypes can hinder detection, making classification and separation more difficult^33,34^. Accurate cell counting requires distinguishing individual objects within clusters, which has been addressed in various contexts, including histopathology and fluorescence microscopy^35–39^.

Detection and segmentation are also essential for cell tracking, where cells or nuclei are typically represented as spots, and their centroids are tracked over time^40^. In this context, precise contour extraction is less critical than accurate centroid localization, which can be achieved using geometric or intensity-based methods^41^. This approach is particularly useful in quantitative studies of cell migration and proliferation, where robust detection in challenging imaging conditions is crucial.

## Results

The evaluation was conducted in two stages. First, the performance of contour detection was assessed using annotated nuclear centroids from fluorescence images as ground truth. Second, a Gaussian-based classification model was used to estimate the total number of cells per image based on the filtered contours.

For image-level cell count prediction, the model was applied to each detected contour, which had been previously filtered for area and shape. Each contour was assigned to one of the four predefined categories (1–4 cells) using the probabilistic model, and these predictions were summed across each image to yield a total estimated cell count.

Prediction accuracy was evaluated using relative tolerance thresholds of ±2.5%, ±5%, and ±7.5% with respect to the ground truth counts derived from fluorescence data. Table 1 summarizes performance for both tasks: contour detection and image-level cell counting.

**Table 1:** Summary of performance metrics for contour detection and image-level cell count estimation.

**Task**	**F1-score**	**Recall**	**Precision**
Contour detection	0.93	0.93	0.94
Cell count ±2.5%	0.69	0.71	0.67
Cell count ±5.0%	0.84	0.86	0.83
Cell count ±7.5%	0.93	0.94	0.92

The first row confirms that the proposed preprocessing pipeline enables reliable contour detection across bright-field microscopy images. The pipeline effectively suppresses small debris, high-frequency noise, and illumination artifacts, resulting in robust contour extraction under challenging imaging conditions.

The subsequent rows indicate that the Gaussian-based classification model performs well in estimating total cell counts, especially when allowing for a relative tolerance of 10–15%, which corresponds to acceptable deviations in practical settings. Additional shape descriptors, including aspect ratio and circularity, were evaluated during model development but did not provide additional discriminative value for estimating cell number and were therefore not included in the final model.

**Fig. 3:**
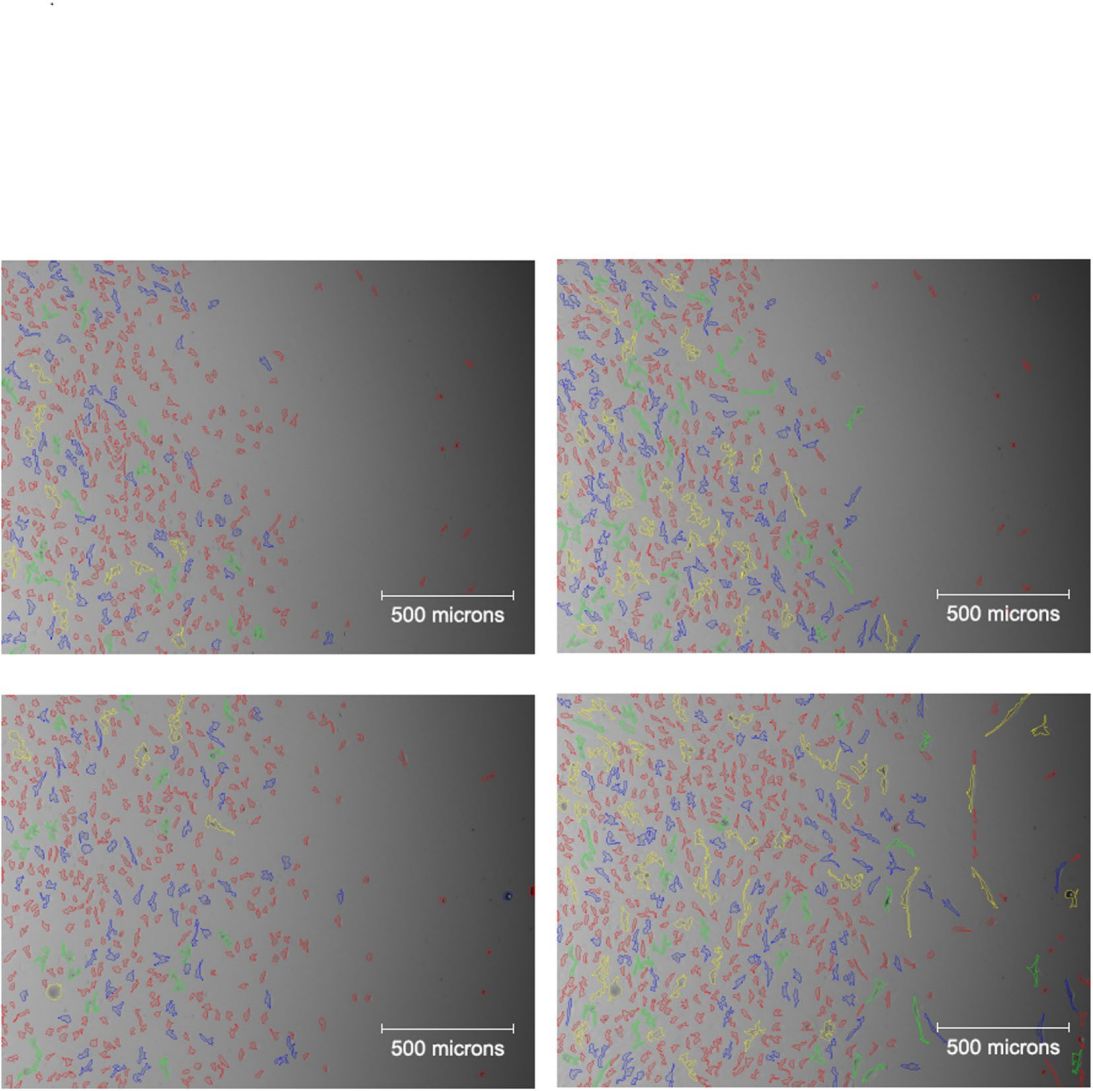
Representative classification of contours by the Gaussian model. Colors indicate predicted cell-count categories: red (1 cell), blue (2 cells), green (3 cells), yellow (4 cells).

Visual classification results in Fig. 3 illustrate the behavior of the probabilistic model across representative images. The image shows correctly classified contours, colored by predicted category: red for one cell, blue for two, green for three, and yellow for four. This visual representation serves as a qualitative check of size-to-category assignment and is not part of the output of the cell counting process itself.

These results confirm that the pipeline enables contour detection and coarse cell count estimation directly from bright-field images. By removing the need for fluorescence labeling or chemical staining, the approach provides a practical, non-invasive solution suitable for label-free time-lapse microscopy analysis.

## Discussion

Over the past years, various approaches have been proposed for the detection, segmentation, or counting of cells in microscopic images, ranging from classical image processing methods to deep learning-based techniques. Classical algorithms based on edge detection (e.g., Canny) combined with morphological operations are still effective for delineating cell areas in bright-field images, although they require careful parameter tuning for elongated or poorly defined cell shapes such as fibroblasts^42^. More advanced techniques employ deformable contour models; for instance, a recently proposed self-initialized level-set algorithm has demonstrated robust performance in segmenting cells even in the presence of significant intensity artifacts and noise^43^.

Results from the Cell Tracking Challenge^44^ demonstrated that classical and hybrid image processing methods can still achieve highly competitive segmentation performance. For example, the KTH-SE algorithm uses purely classical techniques, including Gaussian bandpass filtering followed by seeded watershed segmentation, without any machine learning involved. Similarly, BGU-IL applies adaptive thresholding and morphological operations. These highlight that while deep learning dominates cell detection in microscopy data (particularly when large annotated datasets are available), optimized classical methods still offer competitive performance and should not be overlooked in biomedical applications. Nevertheless, the current trend is shifting toward deep learning, particularly convolutional neural networks (CNNs) such as U-Net architectures^45,46^. One of the few studies addressing the precise segmentation of fibroblasts using deep learning is the work by Roszkowiak et al.^47^, who compared U-Net and LinkNet architectures on time-lapse bright-field images where fibroblasts were relatively well visible and clearly delineated.

In the case of highly noisy, low-contrast, or densely populated images, methods that are not directly based on precise segmentation are often used for cell quantification and phenotypic classification. Ferreira et al.^48^ employed CNN to directly estimate cell counts from unlabeled high-content bright-field microscopy images. Their model successfully learned to quantify cell density for certain lines (e.g., epithelial A549 cells) but struggled with fibroblasts (NIH-3T3 line). This study revealed the challenges associated with counting elongated fibroblasts and highlighted the need for high-quality data when applying CNN-based methods. In a subsequent study, Ferreira and Silveira^49^ extended CNN models to simultaneously classify cell lines and estimate cell counts from bright-field images. While the 3T3 fibroblast line was recognized by the neural network with very high accuracy (>95%), the accurate quantification of their number remained problematic, with 3T3 cells showing the highest prediction error among the tested cell types. These findings confirm that while deep learning models can effectively classify cell populations based on morphology in bright-field images, precise counting of highly elongated cells may still require further methodological improvements.

Our approach is based on extracting object contours from bright-field time-lapse microscopy images using classical image processing techniques, followed by the statistical classification of these contours. Unlike segmentation-based methods, the proposed strategy does not rely on pixel-precise delineation but operates at the contour level, making it more robust to noise, low contrast, and irregular cell morphology. This makes it particularly suitable for challenging bright-field datasets, where cell boundaries are poorly defined and frequently overlap.

In this context, contour classification based on Gaussian-distributed area statistics proved effective. The probabilistic formulation reduces sensitivity to rigid decision boundaries and enables smooth transitions between categories. Additional shape descriptors, including aspect ratio and circularity, were evaluated during model development but did not provide additional discriminative value for estimating cell number in fibroblast-like cell images. This likely reflects the highly variable elongated morphology of fibroblast-like cells, where a single stretched cell and two touching or partially overlapping cells may exhibit similar shape characteristics, particularly during collective migration. In contrast, contour area provided a more stable cue, as the total projected size generally increased with the number of enclosed cells.

Image-level evaluation using relative tolerance thresholds (±5%, ±10%, ±15%) showed that a tolerance of 10–15% is sufficient for practical estimation. Most predictions clustered around zero error, indicating that the model produces unbiased and stable results, while the 5% tolerance proved overly strict for the intended level of approximation. The observed tolerance of 10–15% should be interpreted in relation to the intended application. In practice, this level of tolerance is appropriate for screening and exploratory applications, such as longitudinal monitoring of cell culture dynamics, wound-healing or migration assays^50^, and high-throughput evaluation of biomaterials or soluble factors. From a broader perspective, precision limits on the order of 15% are commonly used as fit-for-purpose criteria in assay validation frameworks^51,52^, and comparable levels of variability have been reported in validated cell-based assays^53^. While these standards do not directly define requirements for cell counting accuracy, they support the interpretation that a 10–15% tolerance is appropriate for screening-level quantitative analyses.

Beyond cell counting, the extracted contours can also serve as a basis for subsequent phenotypic analysis. Recent work by Khang et al.^54^ emphasizes the importance of quantitative morphological descriptors for characterizing fibroblast phenotypes. This suggests that contour-level representations can be further utilized by downstream analytical methods, thereby extending the practical value of the proposed approach beyond counting alone.

Bright-field images are inherently prone to various artifacts; therefore, the proposed pipeline incorporates multiple steps to mitigate their impact. In particular, CLAHE and Wiener filtering proved essential for reliable contour detection. Omitting Wiener filtering resulted in the complete failure of contour detection, while removing other components led to a substantial degradation in performance (see Supplementary Fig. S1 and Supplementary Table S1 online). Although the exclusion of ICA resulted in higher local performance (F1 = 0.95), the overall end-to-end result was inferior (F1 = 0.90), confirming that all components are necessary for consistent performance.

Small debris and high-frequency noise are suppressed using Wiener filtering together with a minimum area threshold (70 pixels), which removes small or spurious structures unlikely to correspond to meaningful cell contours. Uneven illumination is addressed through a combination of ICA, CLAHE, and histogram equalization, which normalizes the background and reduces the likelihood of illumination gradients being detected as contours. As in most label-free approaches, artifact-related detections cannot be completely eliminated, and occasional false positives may still occur. However, the high detection performance (average F1-score of 0.93) indicates that such misclassifications are limited and that the pipeline effectively discriminates between biological structures and common imaging artifacts.

The modular design of the image processing pipeline enables adaptation to diverse imaging conditions and cell types facing similar challenges. The preprocessing steps are primarily tuned to the optical characteristics of the imaging system (e.g., noise and illumination) and therefore remain largely stable across different cell types acquired under similar conditions. At the same time, the classification stage adapts automatically to the analyzed dataset. The Gaussian model parameters (median and standard deviation) are estimated directly from the data, allowing the distributions to shift according to the characteristic size of the cell population. While some parameter adjustments may be required, these are expected to be minimal; in practice, only basic constraints, such as minimum and maximum contour area, may need to be adjusted to match the scale of other spindle-shaped cell types with different average sizes. In addition, decision thresholds between cell-count categories are determined using particle swarm optimization (PSO), allowing classification boundaries to be automatically adapted to a given dataset. Because the method is fully deterministic and requires no annotated training data, transfer to other cell types is straightforward.

The approach was motivated by practical requirements in laboratory studies of fibroblast and fibroblast-like synoviocyte migration and proliferation, where reliable and scalable analysis of label-free bright-field data is needed. In this context, the pipeline addresses a specific but practically relevant gap between classical image processing and robust quantitative analysis. While the current study focuses on a controlled experimental setup, more complex scenarios involving highly dense or heterogeneous cell populations remain an important direction for future work. Overall, the results confirm that combining noise-resilient preprocessing with a Gaussian-based contour classification model yields an effective and interpretable solution for high-throughput bright-field microscopy analysis without relying on fluorescent labeling or annotated training data.

## Methods

### Dataset

We present our method using a dataset of bright-field time-lapse microscopy images, accompanied by corresponding fluorescence images. The dataset consists of images of fibroblast-like synoviocytes obtained from the knee of a patient with osteoarthritis, acquired using a BioTek Cytation 5 Cell Imaging Multimode Reader equipped with a Blackfly BFLY-U3-23S6M camera (serial number: 19069051). The acquisition parameters were configured as follows: saturation level of 65.504$\upmu$m; camera gain of 0.1; brightness level of 50; contrast level of 33; focal height of 2539.2$\upmu$m; focal metric ratio of − 1; bottom elevation of 2539.2$\upmu$m; and LED intensity of 4. Images were captured at a resolution of 96 dpi, with a time interval of 20 min between frames, over a total duration of 66 h and 40 m.

**Fig. 4:**
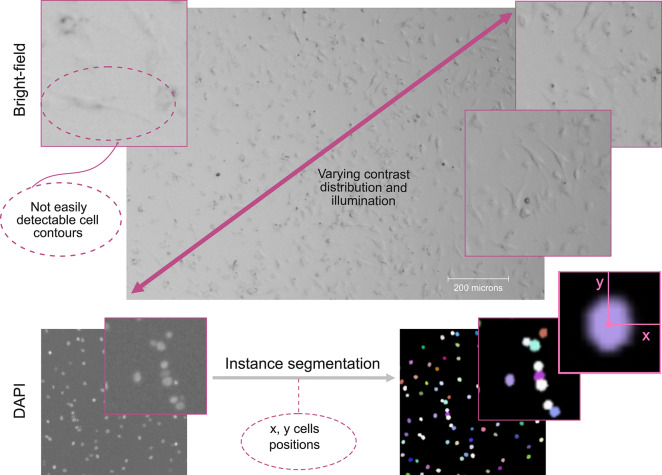
Microscopic images of fibroblasts acquired using a time-lapse automated microscope. Each frame was captured in two channels: a low-contrast bright-field image and a fluorescence image highlighting cell nuclei stained with Hoechst dye. While the nuclei of fibroblasts can be easily segmented using available computational tools–even by non-expert users (e.g., the StarDist tool). The bright-field images suffer from uneven illumination, noise, artifacts, and poorly distinguishable cell contours. These factors complicate segmentation using both traditional image processing methods and deep-learning approaches (e.g., U-Net).

We evaluated 2 datasets, each consisting of 200 bright-field images (used for method development and testing) and 200 corresponding fluorescence images acquired with a DAPI filter set after staining nuclei with Hoechst 33342. The centroids of the detected nuclei were used as ground truth for validation. The use of two distinct datasets enables a more comprehensive assessment of the robustness and generalizability of the proposed image processing pipeline, helping to ensure that the method is not overfitted to a single experimental condition. The specific characteristics of our microscopic image dataset of fibroblast-like synoviocytes are illustrated in Fig. 4.

#### Nuclei detection method

Individual cell nuclei visible in the fluorescence images were identified using the StarDist detector implemented in Fiji/ImageJ, which performs instance segmentation^55^. The algorithm employs a lightweight neural network based on the U-Net architecture and operates similarly to most detection methods. However, instead of predicting axis-aligned bounding boxes, it represents shapes by predicting star-convex polygons for each pixel. In our workflow, the output of the StarDist plugin in Fiji/ImageJ is a .csv table containing the *x* and *y* coordinates of the detected nuclei within the image. These coordinates (nuclei centroids) were subsequently used as ground truth locations for validating the bright-field detection method.

### Contour detection workflow

Accurate cell detection in low-contrast microscopy requires a precise image conditioning pipeline. This section outlines a step-by-step process that systematically suppresses noise, enhances structural features, and isolates contours corresponding to fibroblast cells. Each operation is tailored to maximize signal clarity while preserving fine details essential for downstream analysis.

The following pipeline transforms raw input images into contour candidates suitable for further classification. The sequence includes statistical separation, local and global contrast enhancement, noise suppression, structural boosting, and final morphological shaping. The entire process is designed for computational efficiency and robustness across varying imaging conditions.

#### Image processing pipeline for contours extraction

**Fig. 5:**
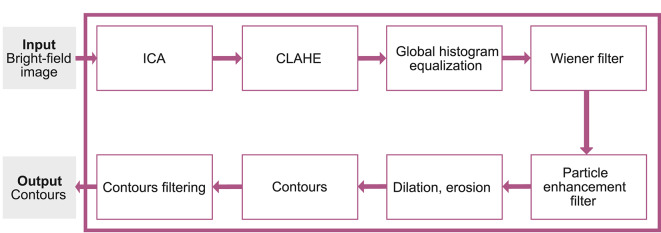
Pipeline for contour extraction.

Figure 5 shows the complete image processing pipeline for contour extraction. Each operation builds on the previous one, enhancing the signal-to-noise ratio and emphasizing features associated with cell morphology.

1. *Independent component analysis (ICA):* ICA isolates statistically independent structures from noise and background interference. This step enhances biological features and suppresses overlapping signals that obscure cell contours^56,57^.
2. *Contrast limited adaptive histogram equalization (CLAHE):* CLAHE boosts local contrast without over-amplifying homogeneous regions. This adaptive method highlights weakly defined structures and prepares the image for global tuning^58,59^.
3. *Global histogram equalization:* Global intensity redistribution complements CLAHE by enhancing the full dynamic range. This step balances local and global contrast for improved visibility of cellular shapes^60,61^.
4. *Wiener filter:* An adaptive filter that reduces residual noise while preserving edges. It smooths homogeneous areas without blurring structural boundaries, preparing a clean input for contour isolation^60,62^.
5. *Particle enhancement filter (PEF): *PEF selectively amplifies fine, particle-like structures such as cell edges. It boosts relevant regions and increases the separation between cells and background artifacts. More details about the author’s method are in^63^.
6. *Morphological operations: dilation, erosion, and contour extraction:* Morphological operations finalize shape preparation. Dilation bridges small gaps and connects nearby segments. Erosion removes noise and sharpens features. Contour extraction isolates and traces object boundaries^64–66^.
7. *Contour filtration:* Detected contours are filtered based on area and shape. Non-relevant structures are discarded, and the remaining contours provide a preliminary estimate of cell count.

The preprocessing steps were designed to be computationally efficient, allowing real-time or near real-time processing of microscopy images. On hardware (AMD Ryzen 7 4800H with 16GB RAM), the complete pipeline processes a single image in approximately 1.2 s. For a sequence of 200 images, the total processing time is around 4 min, demonstrating that the method remains scalable and suitable for high-throughput analysis. These results indicate that our approach can efficiently handle large datasets without significant computational overhead, making it practical for integration into automated analysis pipelines.

**Fig. 6:**
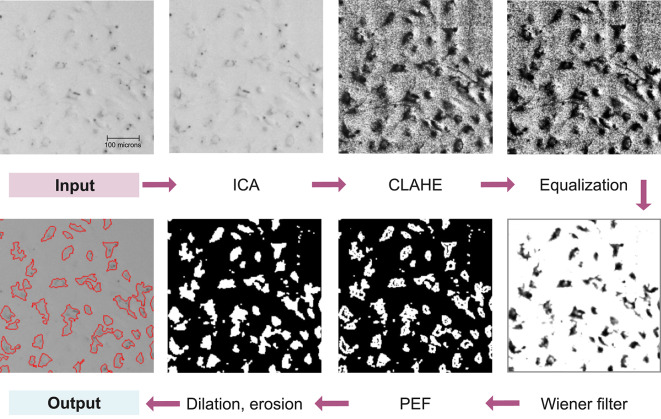
Image processing from loading to getting contours.

An overview of the complete contour detection workflow, from initial image loading to final contour extraction, is illustrated in Fig. 6.

#### Contour detection workflow assessment

The aim of this assessment step is solely to verify whether the contours extracted from bright-field microscopy images correspond to real fibroblast cells. The process does not determine the precise number of nuclei within contours but instead focuses on validating contour positions and detecting false positives and false negatives. Fluorescently annotated nuclear centroids serve as ground truth for this validation. A spatial tolerance of ±5 pixels was used to accommodate minor positional discrepancies between fluorescence and bright-field images. The output of this assessment is expressed in terms of F1-score, precision, and recall, quantifying the algorithm’s ability to reliably isolate biologically relevant contours while minimizing false detections.

To classify detection outcomes, the following deterministic scheme was applied:

- *True Positive (TP):* A nuclear coordinate that is located within the boundary of a detected contour.
- *False Positive (FP):* A contour does not contain any nuclear coordinate.
- *False Negative (FN): *A nuclear coordinate that is not enclosed by any detected contour.

**Fig. 7:**
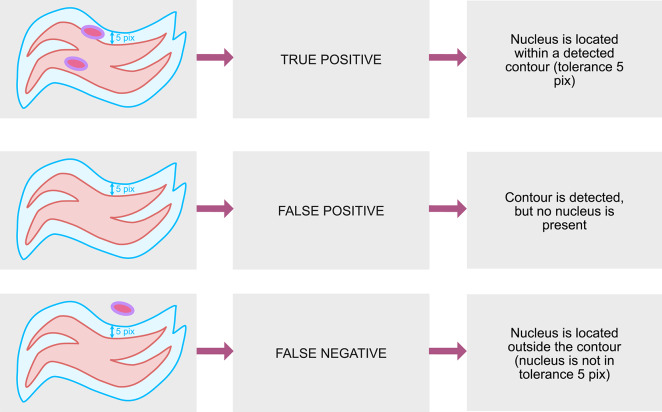
Illustration of contour validation criteria. A detection is classified as a true positive if a nucleus is located within the boundary of a detected contour. A true positive is also assigned when the nucleus lies outside the contour but remains within a predefined tolerance region. A false positive is recorded when a contour is detected without any corresponding nucleus inside. Finally, if a nucleus is located outside both the contour and the defined tolerance region, the outcome is classified as a false negative.

Each nucleus was matched to a single contour. Overlapping or duplicate detections were excluded. The classification scheme is illustrated in Fig. 7, which visualizes all three possible outcomes of the validation process.

Intermediate outputs were inspected after each preprocessing step (ICA, CLAHE, histogram equalization, Wiener filtering, particle enhancement) to verify progressive contour enhancement and noise suppression. Final morphological operations (dilation, erosion) improved edge integrity and removed spurious artifacts. Contours were also visually compared with expected fibroblast morphology to ensure shape consistency.

Quantitative performance was assessed using the F1-score, computed from aggregated TP, FP, and FN. Precision and recall were derived accordingly. This approach confirmed that each step of the pipeline contributed to accurate and reproducible cell identification.

**Fig. 8:**
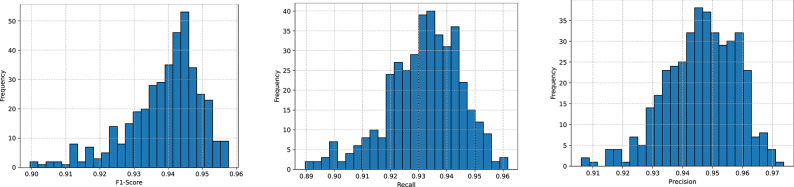
Distributions of F1-score, recall, and precision across 400 bright-field microscopy images. All metrics show tight variance, indicating consistent and robust detection performance.

To further assess robustness, detection metrics were computed across a large validation set of 400 images, and their distributions were analyzed. Histograms of F1-score, precision, and recall (see Fig. 8) show consistent performance across the dataset with low variability and absence of outliers.

Detection performance across 400 images exhibited low variability, as reflected in the metric distributions summarized in Table 2.

**Table 2:** Contour detection metrics summary across 400 images.

**Metric**	**Min**	**Max**	**Global average**
F1-score	0.90	0.96	0.93
Recall	0.89	0.96	0.93
Precision	0.91	0.97	0.94

**Fig. 9:**
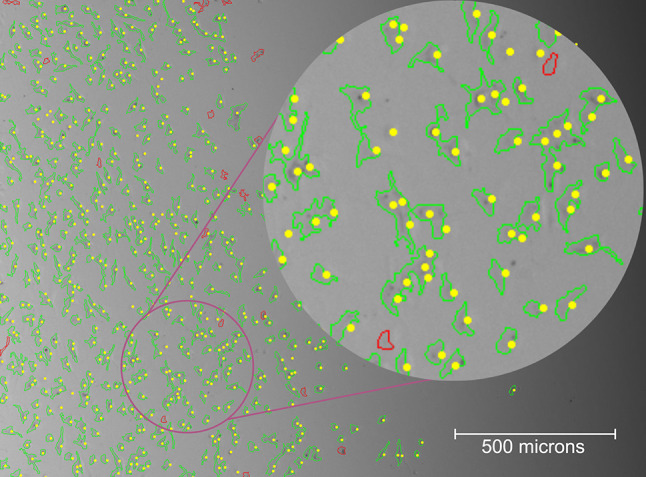
Examples of validation outputs illustrating true positives (green), false positives (red), and false negatives (yellow). Yellow dots indicate the *x,y* coordinates of ground truth nuclei centroids. Green contours represent correctly detected cell contours containing the appropriate number of nuclei, while red contours correspond to incorrectly detected cell contours that do not contain any nucleus.

The pipeline maintained consistent F1-scores, precision, and recall across diverse imaging conditions, confirming its robustness and suitability for large-scale contour detection. Representative validation outputs with classified contours (TP, FP, FN) are shown in Fig. 9.

However, accurate contour detection alone does not provide information about the exact number of fibroblast cells enclosed within each contour. Therefore, the next step focuses on quantifying the cell counts within these detected contours, which is addressed in detail in the following section.

### Contour-based cell quantification

Having extracted contours corresponding to fibroblast cells, the next objective was to estimate the number of cells enclosed within each detected contour. This was achieved by constructing a probabilistic classification model based on Gaussian distributions derived from annotated fluorescence images.

For each filtered contour, the number of enclosed nuclei was determined by comparing its geometry with nuclear centroid coordinates obtained from the fluorescence channel, using a spatial tolerance of ±5 pixels to account for minor misalignments. Based on this count (1–4), each contour was assigned to a corresponding category. The contour area was then recorded and grouped according to these categories, resulting in four distributions.

These distributions exhibit right-skewed behavior consistent with approximately log-normal characteristics, reflecting biological variability in cell size and aggregation into clusters. To model these data, Gaussian probability distributions were fitted to each group, using the median and standard deviation as robust estimates of central tendency and dispersion. Rather than assuming strict normality, the Gaussian model serves as a local approximation of the central region of each distribution.

**Fig. 10:**
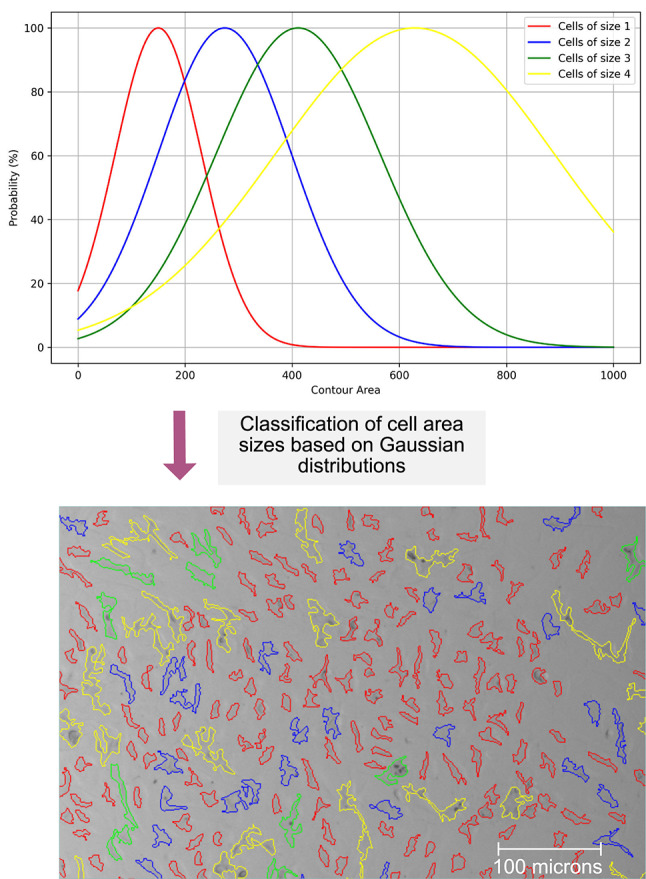
Overlapping Gaussian distributions for cell-count categories (1–4) derived from contour area statistics. Contours are classified by the most probable category and visualized accordingly in the sample image. Smooth probability transitions help reduce ambiguity near decision boundaries.

The distributional properties were further assessed using formal normality testing and Quantile–Quantile (Q–Q) plots of log-transformed data (seeFig. S2 online). This model enables classification of new contours by selecting the most probable category based on area. Overlapping Gaussian curves ensure smooth transitions between classes and reduce ambiguity near decision boundaries. The resulting distributions and corresponding contour classifications are visualized in Fig. 10.

Two correction rules were introduced to refine classification in ambiguous regions:

- If a contour’s area lay between the medians of the one-cell and two-cell distributions and the probability difference was $\le$ 0.5, it was assigned to the one-cell category.
- If it lay between the medians of the two-cell and three-cell distributions and the probability difference was $\le$ 0.25, it was assigned to the two-cell category.

**Fig. 11:**
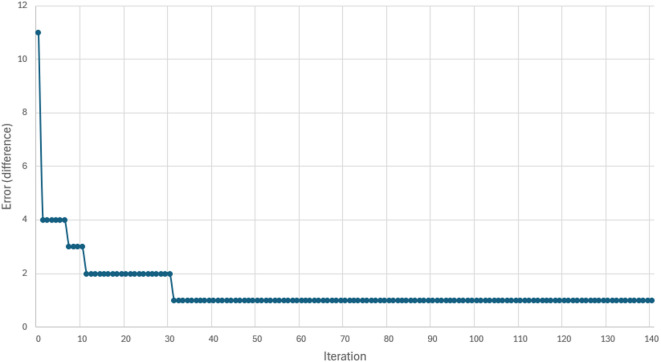
Convergence of classification error during Particle Swarm Optimization. The algorithm tuned the probability thresholds for category boundaries to balance precision and recall.

The thresholds for these rules were optimized using Particle Swarm Optimization (PSO) to minimize classification error across the dataset. PSO was selected due to the non-differentiable and potentially non-convex nature of the objective function arising from discrete cell counting and non-linear Gaussian modeling. The probability thresholds ($\Delta P_{1-2}$ and $\Delta P_{2-3}$) were defined within bounded ranges: $\Delta P_{1-2} \in [0.10, 0.48]$ and $\Delta P_{2-3} \in [0.15, 0.38]$. The PSO was implemented using the GlobalBestPSO optimizer from the pyswarms library with the following hyperparameters: cognitive coefficient $c_1 = 2.2$, social coefficient $c_2 = 2.5$, and inertia weight $w = 0.85$. The swarm consisted of 50 particles and was run for 140 iterations. A fixed random seed (np.random.seed(42)) was used to ensure reproducibility. Convergence of the optimization process is shown in Fig. 11.

### Image-level cell count evaluation

The final evaluation was performed by aggregating the predicted number of cells per image and comparing it with fluorescence-derived ground truth counts. Each filtered contour was classified into a cell-count category (1–4 cells) using the Gaussian-based model, and the total number of predicted cells per image was obtained as the sum of these contour-level estimates.

Rather than using a fixed numerical threshold, prediction accuracy was assessed using a relative tolerance defined as a percentage of the true image-level cell count. Three tolerance levels were evaluated: 5%, 10%, and 15%, which correspond to symmetrical intervals (±2.5, ±5.0, and ±7.5 percent) around the true count. A prediction was classified as a true positive (TP) if it fell within the allowed interval, a false positive (FP) if it exceeded the upper bound, and a false negative (FN) if it was below the lower bound.

**Fig. 12:**
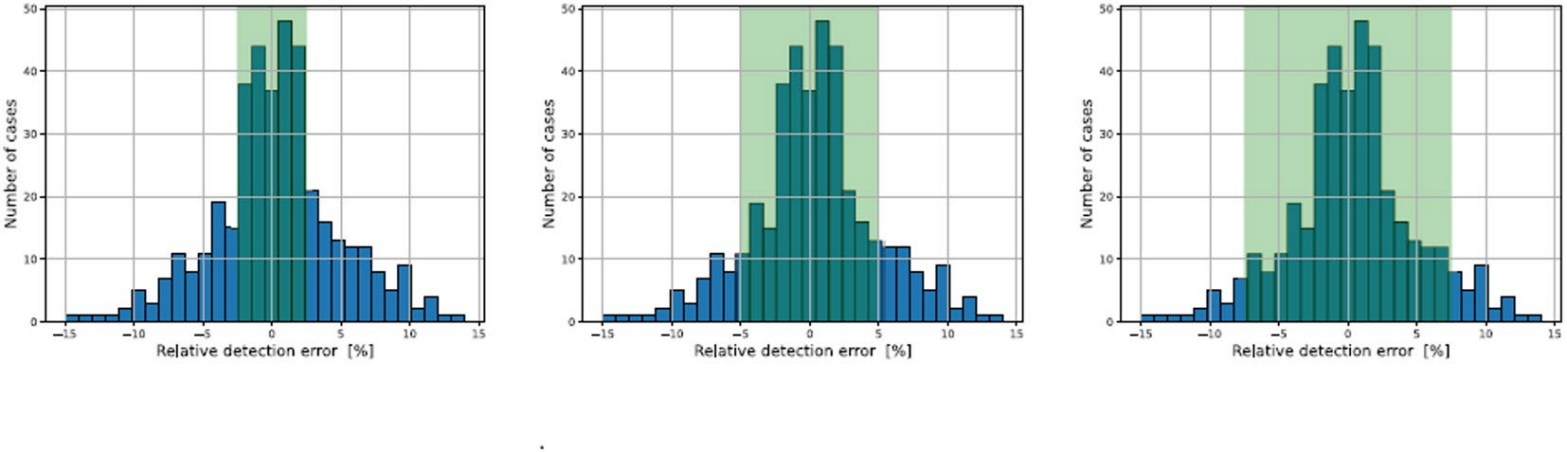
Distribution of relative detection errors for tolerance thresholds of 5%, 10%, and 15%. The green areas indicate acceptable deviation ranges around the true cell counts.

The distribution of prediction errors for all three tolerance levels is visualized in Fig. 12. Most predictions clustered around zero error, indicating stable estimation centered on the ground truth.

The summary of performance metrics in Table 3 demonstrates that the most practical tolerance levels are 10% or 15%, where the F1-score reached 0.84 and 0.93, respectively. In contrast, the 5% threshold proved overly restrictive and resulted in lower accuracy (F1 = 0.69). Given the objective of estimating image-level cell counts without fluorescence labeling, a 10–15% tolerance provides a robust and biologically meaningful approximation.

**Table 3:** Image-level performance metrics for different tolerance thresholds.

**Metric**	Tolerance ±2.5 %	Tolerance ±5.0 %	Tolerance ±7.5 %
F1 Score	0.69	0.84	0.93
Recall	0.71	0.86	0.94
Precision	0.67	0.83	0.92

## Supplementary Information

Supplementary Information.

## Data Availability

The dataset supporting the findings of this study is publicly available at Zenodo (https://doi.org/10.5281/zenodo.18817576), along with the source code required to ensure full reproducibility. The source code is also maintained on GitHub at https://github.com/MartinBrigada/Fibroblast-detection-low-contrast/tree/main.
