# Supplementary material for: A deterministic method for quantifying spindle-shaped cells in noisy bright-field microscopy
Source: Sci Rep. 2026 May 21;16:23275. doi: 10.1038/s41598-026-51592-x (PMC13402725; doi:10.1038/s41598-026-51592-x)
Supplement: Supplementary file 1 — Supplementary Information. [file 41598_2026_51592_MOESM1_ESM.pdf]

Supplementary Information

A deterministic method for quantifying spindle-shaped cells in noisy bright-field microscopy

Martin Radvanský[1], Markéta Vašinková[1,*], Miloš Kudělka[1], Eva Kriegová[2] & Petr Gajdoš[1]

[1]VSB – Technical University of Ostrava, Department of Computer Science, FEECS, 17. listopadu 2172/15, Ostrava, 708 00, Czech Republic

[2]Faculty of Medicine and Dentistry, Palacký University & University Hospital, Department of Immunology, Hněvotínská 976/3, Olomouc, 775 15, Czech Republic

[*]Correspondence: marketa.vasinkova@vsb.cz

Configuration	Metric	Min	Max	Mean
Full pipeline (proposed)	F1 score	0.90	0.96	0.93
	Recall	0.89	0.96	0.93
	Precision	0.91	0.97	0.94
Without CLAHE	F1 score	0.73	0.94	0.83
	Recall	0.63	0.95	0.79
	Precision	0.85	0.95	0.90
Without Wiener filter	F1 score	0.00	0.00	0.00
	Recall	0.00	0.00	0.00
	Precision	0.00	0.00	0.00

Table S1: Ablation study of preprocessing components. The full pipeline achieved consistently high performance across all evaluated metrics. Removing CLAHE reduced recall and the minimum F1 score, indicating lower robustness in low-contrast regions. Exclusion of the Wiener filter resulted in complete failure of contour detection due to noise-dominated responses.

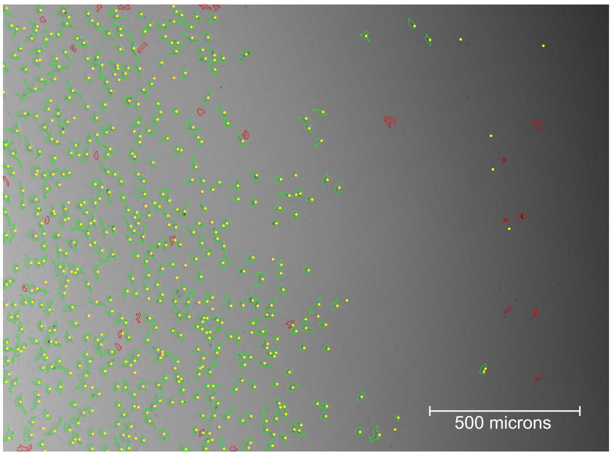

(a)

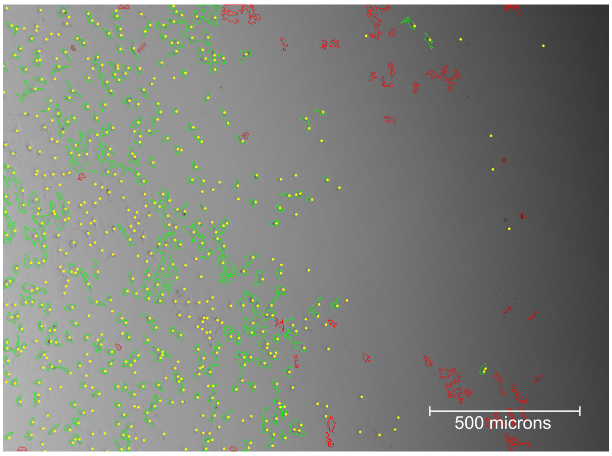

(b)

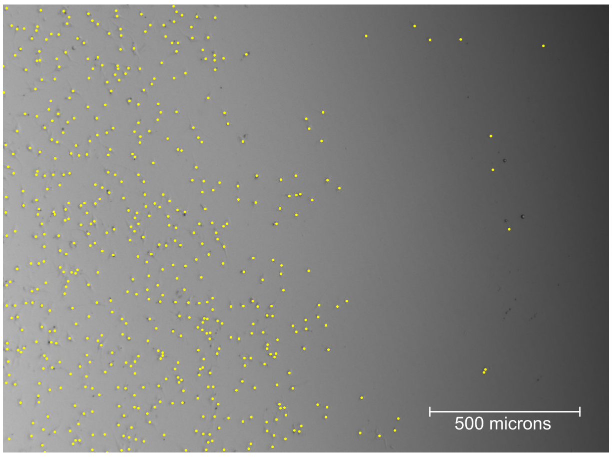

(c)

Figure S1: Impact of preprocessing components on contour detection performance.(a) Full preprocessing pipeline used in the proposed method, showing robust contour extraction under noisy bright-field conditions, (b) Pipeline without CLAHE, resulting in reduced sensitivity, particularly in low-contrast regions, (c) Pipeline without Wiener filtering, leading to failure of contour detection due to noise-dominated responses.

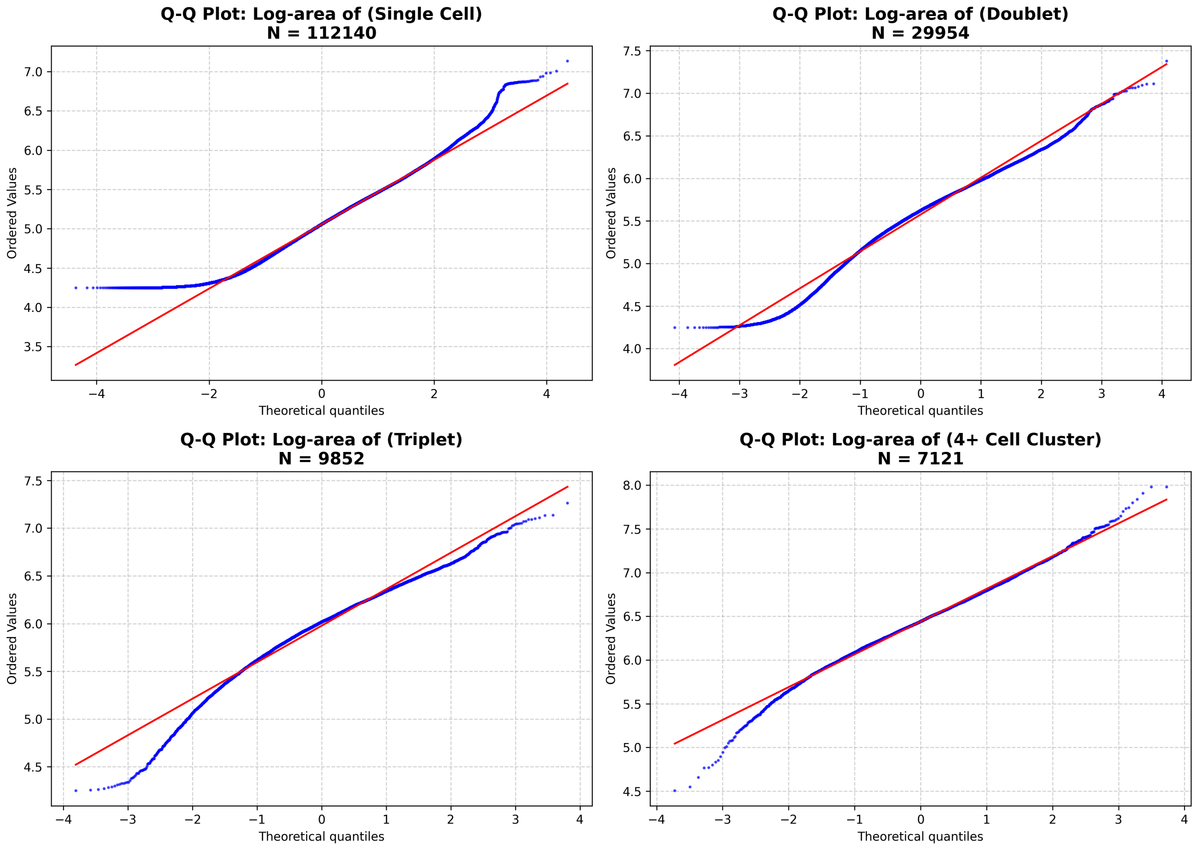

Figure S2: Quantile–Quantile plots of log-transformed contour area distributions. Q–Q plots for all cell-count categories show that the central regions of the log-transformed distributions follow an approximately normal trend, supporting the use of a Gaussian approximation for probabilistic classification.
